# Low Back Pain, Physical Activity, and Muscle-Strengthening Activity Among Saudi Adults: A Cross-Sectional Study

**DOI:** 10.3390/healthcare14213571

**Published:** 2026-10-22

**Authors:** Albatool Humod Alnojeidi

**Affiliations:** Department of Family and Community Medicine, College of Medicine, Al Imam Mohammad Ibn Saud Islamic University (IMSIU), Riyadh 13318, Saudi Arabia; ahalnojeidi@imamu.edu.sa; Tel.: +966-505989823

**Keywords:** low back pain, physical activity, muscle-strengthening activity, physical activity guidelines, Saudi Arabia, cross-sectional study

## Abstract

**Highlights:**

**What are the main findings?**

- There was no clear evidence that meeting aerobic physical activity thresholds (combined, moderate-intensity, or vigorous-intensity; walking not counted) was associated with chronic low back pain among Saudi adults.
- Meeting the muscle-strengthening frequency threshold (≥2 days/week) was associated with lower odds of chronic low back pain (aOR = 0.606; 95% CI, 0.385–0.954), a statistically borderline result. The estimate for 1 day/week did not differ significantly from no activity or from ≥2 days/week.

**What are the implications of the main findings?**

- Aerobic and muscle-strengthening activity may relate to low back pain differently. This study did not test that difference directly, so it is a question for further research and not a basis for changing advice.
- The muscle-strengthening finding is preliminary because of the cross-sectional design and self-selected online sample, and it needs testing in longitudinal studies.

**Abstract:**

**Background/Objectives**: Physical activity (PA) and muscle-strengthening activity (MSA) are recommended for health, but their association with low back pain (LBP) is inconsistent and has rarely been examined against guideline thresholds in Saudi adults. This study examined whether meeting aerobic PA thresholds and the MSA frequency threshold was associated with chronic LBP (≥3 months). **Methods**: In this cross-sectional online survey (September 2025 to April 2026), 449 Saudi adults were analyzed. Exposures were meeting the thresholds for combined moderate-to-vigorous PA (≥150 moderate-equivalent minutes/week, walking not counted), moderate-intensity PA (≥150 min/week), vigorous-intensity PA (≥75 min/week), and MSA frequency (≥2 days/week; also modeled as 0, 1, and ≥2 days/week). Logistic regression adjusted for age, sex, and body mass index estimated adjusted odds ratios (aORs). **Results**: Chronic LBP was reported by 239 participants (53.2%). No statistically significant association was detected for combined PA (aOR = 0.904; 95% CI, 0.599–1.364), moderate-intensity PA (aOR = 0.813; 95% CI, 0.532–1.244), or vigorous-intensity PA (aOR = 0.935; 95% CI, 0.618–1.416). Meeting the MSA frequency threshold was associated with lower odds of LBP (aOR = 0.606; 95% CI, 0.385–0.954; *p* = 0.031). In the three-level model, 1 day/week did not differ significantly from no MSA (aOR = 0.949; 95% CI, 0.515–1.748) or from ≥2 days/week. **Conclusions**: There was no clear evidence of an association between aerobic PA thresholds and chronic LBP, although power may have been limited. Meeting the MSA frequency threshold was associated with lower odds of LBP, but the estimate is statistically borderline and the data do not show a frequency threshold. Given the cross-sectional design and self-selected sample, this finding is preliminary and needs longitudinal confirmation.

## 1. Introduction

Low back pain (LBP) is a highly prevalent condition that affects up to 70% of people worldwide [1,2]. It is often accompanied by physical and functional impairments, reduced quality of life, and substantial psychological and social consequences [3,4,5]. This burden is particularly relevant to the Middle East and North Africa, which have the second-highest LBP prevalence worldwide (14.8%) [2]. In Saudi Arabia, a published meta-analysis of 124 studies estimated the 12-month prevalence of LBP at 52.3% and lifetime prevalence at 64.0% [6]. An unpublished systematic review by the author found a similar pooled prevalence (63.5%) across occupational, educational, and community populations and pointed to obesity, physical inactivity, smoking, psychological distress, and occupational exposure as factors that could plausibly be changed [7].

LBP rarely has a single cause. Biology, biomechanics, behavior, psychology, and occupational exposure all play a part, and they interact rather than act separately [3]. Physical activity (PA) is one of the few modifiable factors, with well-documented benefits for health and physical function [3]. Current guidelines recommend 150 min a week of moderate-intensity PA, 75 min of vigorous-intensity PA, or some equivalent mix, plus muscle-strengthening activity (MSA) at least twice a week [8,9]. Whether meeting these targets is associated with lower odds of LBP remains an open question.

The PA-LBP relationship does not appear to be a straightforward dose–response curve. Some work has tied insufficient PA to higher LBP risk [10]; a meta-analysis covering nearly 96,000 adults found moderate total and leisure-time PA linked to a 10% lower risk, but no extra benefit beyond that level [11]. Results also shift with the type and amount of activity measured, and one cross-sectional study raised the possibility that the relation is U-shaped, with more chronic LBP among both sedentary adults and those doing physically strenuous activity [12]. The MSA appears different again. Strengthening exercise works through muscular capacity and spinal function rather than aerobic conditioning [13,14]. In a US sample, adults meeting MSA guidelines had lower odds of LBP [15].

This relationship is particularly relevant in the Saudi context; the 2025 Saudi Health Report classified 41.5% of adults as insufficiently active [16], and LBP already carries a heavy population burden [6,7]. Saudi research on LBP has mostly estimated prevalence or listed correlates one at a time [6,7]. Where activity was examined, it was usually recorded as general exercise habits, a change in activity, or sitting time [17,18]. To the author’s knowledge, no Saudi study of adults has combined separate comparisons for the non-walking aerobic PA threshold (≥150 moderate-equivalent minutes/week) and the MSA frequency threshold (≥2 days/week) with a self-reported chronic LBP outcome. This combination is what the present study adds. In this study, LBP was restricted to symptoms lasting ≥3 months to isolate a persistent, clinically meaningful condition, rather than a passing episode [19,20], where its relationship with habitual behaviors such as PA and MSA may differ from that of acute LBP.

The PA guidelines set separate aerobic and muscle-strengthening targets [8,9], and there are reasons to expect the two to relate to LBP differently. Aerobic PA is counted as total minutes, which mixes activities that load the spine in different ways, and pooled observational estimates for PA and LBP are modest and inconsistent [10,11]. MSA targets muscle strength and endurance, the capacities that exercise programs for LBP aim to build. A review of exercise for existing chronic LBP reported benefit from programs that build muscular strength [13], prevention trials found a lower risk of LBP with exercise programs, most of which combined strengthening with stretching or aerobic exercise [14], and a US population study found lower odds of LBP among adults doing MSA on ≥2 days/week [15]. This study therefore examined the association between meeting international aerobic PA and MSA recommendations and chronic LBP (≥3 months) among Saudi adults, and analyzed the two exposures separately. Secondary objectives were to examine moderate- and vigorous-intensity PA separately, and whether sex modified the PA/MSA-LBP associations. The primary hypothesis was that meeting the PA and MSA recommendations would each be associated with lower odds of chronic LBP, with the stronger association expected for MSA.

## 2. Materials and Methods

### 2.1. Study Design and Participants

This cross-sectional study was conducted among Saudi adults and reported in accordance with the Strengthening the Reporting of Observational Studies in Epidemiology (STROBE) guidelines [21]. The study was conducted in accordance with the Declaration of Helsinki and was approved by the Institutional Review Board of Al Imam Mohammad Ibn Saud Islamic University (approval No. 739/2024; date of approval: 22 December 2024). Electronic informed consent was obtained from all participants before participation.

Data were collected between September 2025 and April 2026 through an online survey distributed through WhatsApp (Meta Platforms, Inc., Menlo Park, CA, USA) groups and official university email distribution lists; the email distribution was approved and supported by Al Imam Mohammad Ibn Saud Islamic University. This was a convenience sample; no sampling frame was used. Interested individuals were directed to a Google Forms (Google LLC, Mountain View, CA, USA) link that presented the study information and an electronic consent form. After consenting, the participants answered two screening questions to determine eligibility before proceeding to the survey (“Are you a Saudi national?”; “Are you currently pregnant?”). Those who did not qualify were thanked for their time and exited automatically. Age, nationality, pregnancy, and all other eligibility information were self-reported and were not checked against records. LBP attributable to a specific pathology was also self-identified, through a survey question on the cause of the back pain. The survey was anonymous and the form did not limit respondents to one submission. Before data analysis, the dataset was checked for identical records across all survey items, and none were found. Only respondents who passed screening could complete the survey.

Individuals aged ≥19 years were eligible if they were Saudi nationals. Exclusion criteria were pregnancy and LBP attributable to a specific underlying pathology (e.g., fracture, infection, malignancy, inflammatory spinal disease, or other serious spinal conditions). As this study focused on LBP lasting ≥3 months, participants with LBP lasting <3 months (*n* = 81) were excluded from the analytical sample, whereas participants without LBP were included in the comparison group. The outcome of interest was chronic LBP, and the standard criterion for chronic LBP is pain lasting 3 months or longer [19,20]. Chronic LBP, and not acute or subacute LBP, was chosen for clinical reasons. Acute and subacute episodes can be transient: in meta-analyses of cohort studies, pain and disability improved markedly within the first 6 weeks of an episode [22,23], whereas pain that had already lasted 12 weeks or longer stayed at high levels with minimal improvement over the following year [23]. A shorter episode may therefore improve or resolve soon after the survey, and a case group that mixed such episodes with persistent pain would combine conditions with different clinical courses. Chronic LBP is also associated with impaired physical function, mental health, and quality of life [4], and a persistent condition is a more suitable outcome than a passing episode for studying habitual behaviors such as PA and MSA. Respondents with current LBP of shorter duration did not meet the 3-month criterion, and because they had current pain they could not serve as pain-free comparators either. The comparison is therefore between adults with chronic LBP and adults with no current LBP. This exclusion removed 15.3% of respondents and may introduce selection bias, which was examined in a sensitivity analysis (Section 2.4) and is discussed as a limitation.

### 2.2. Sample Size

During data collection, the adequacy of the sample size was assessed to confirm that enough participants would be recruited. The assessment used G*Power (version 3.1.9.7; Heinrich Heine University Düsseldorf, Düsseldorf, Germany) [24], with Fisher’s exact test for two independent proportions, and was based on the study by Gu et al. [25]. In that study, the chronic LBP prevalence was 6.6% among participants meeting PA recommendations (*n* = 211) and 23.8% among those with insufficient PA (*n* = 261). Using these proportions (p_1_ = 0.066, p_2_ = 0.238), a two-sided α of 0.05, statistical power (1 − β) of 95%, and an allocation ratio (*N*_2_/*N*_1_) of 1.24, the ratio of the two group sizes in that study, the minimum required sample size was 224 participants (100 meeting PA recommendations, 124 not meeting them). An additional 15% to allow for incomplete questionnaires, missing data, or invalid responses gave a minimum of 258 participants. The analytical sample of 449 exceeded this figure. The calculation applies to the PA comparison only.

### 2.3. Measures

#### 2.3.1. Low Back Pain (LBP)

The primary outcome was LBP status (yes/no), determined from responses to “Are you currently complaining of low back pain?” and “For how long have you been complaining of low back pain?” LBP was defined as pain located between the lower margin of the ribs and gluteal folds [20]. Chronic LBP was defined by the standard duration criterion, as current LBP lasting ≥3 months [19,20]. The pain-frequency criterion of the National Institutes of Health (NIH) Task Force definition [20] was not collected. Participants reporting LBP for ≥3 months were classified as having LBP, and those not reporting LBP were classified as not having LBP. All LBP cases included in the analyses met the prespecified chronic-duration criterion, and in the Results “LBP” refers to LBP lasting ≥3 months. Respondents with no current LBP were classified as not having LBP whether or not they had LBP in the past; past LBP was not asked. The outcome is the presence of self-reported chronic LBP. Pain intensity, disability, and clinical diagnosis were not assessed.

#### 2.3.2. Physical Activity (PA)

PA during the previous seven days was measured using the official Arabic short form of the International Physical Activity Questionnaire (IPAQ-SF; last 7 days, self-administered) [26,27], which assesses the frequency and duration of vigorous-intensity PA, moderate-intensity PA, walking, and sitting time. The Arabic items were used without changes to their wording. Participants reported days per week and the duration per day of moderate- and vigorous-intensity PA. The IPAQ-SF has demonstrated acceptable reliability and validity across populations and is widely used in epidemiological research to assess population PA levels [28].

Weekly minutes of vigorous- and moderate-intensity PA were calculated by multiplying the frequency (days/week) by duration (minutes/day). Moderate-equivalent PA minutes were calculated by summing weekly moderate-intensity minutes and twice the weekly vigorous-intensity minutes. In accordance with current international recommendations [8], participants were classified as meeting the combined PA recommendation if they accumulated ≥150 moderate-equivalent minutes/week, referred to here as the combined moderate-to-vigorous PA (MVPA) threshold. The formula follows the guideline, which sets the aerobic recommendation as at least 150 min a week of moderate-intensity activity, at least 75 min of vigorous-intensity activity, or an equivalent combination in which one minute of vigorous activity counts as two minutes of moderate activity [8]. Brisk walking counts as moderate-intensity activity under the guideline, but the IPAQ-SF records walking in separate items without pace, so reported walking could not be classified as moderate intensity and was not added to the total. Usual walking time per day was asked as a free-text item. These answers were therefore not used in the analysis. This differs from the IPAQ scoring protocol, which adds walking to its total activity score [26]; the variable used here is a guideline-based MVPA measure and not the IPAQ total score. Adults whose aerobic activity is mainly brisk walking may therefore have been classified as not meeting the threshold. Walking days per week are reported descriptively. Moderate- and vigorous-intensity PA were also evaluated separately using thresholds of ≥150 min/week and ≥75 min/week, respectively. Descriptive statistics for moderate- and vigorous-intensity PA (days/week, minutes/session) were calculated only among participants reporting engagement in that intensity. Participants reporting no activity of a given intensity were coded as zero minutes/week and retained in all regression analyses.

#### 2.3.3. Muscle-Strengthening Activity (MSA)

MSA was assessed using two questions addressing participation during the preceding 30 days: “Over the past 30 days, did you do any physical activities specifically designed to strengthen your muscles, such as lifting weights, push-ups or sit-ups?” and “Over the past 30 days, how many days per week did you typically do these activities?” The response options for the second question were 0 days, 1 day, or 2 or more days a week. The 30-day recall period was used to capture habitual MSA participation, which may occur less frequently than aerobic PA.

Participants were classified as having no MSA (0 days/week), MSA on 1 day/week, or MSA on ≥2 days/week, the frequency set by the current recommendation [8]. The recommendation also specifies moderate or greater intensity and all major muscle groups [8], and the questions did not ask about either. The exposure is therefore the frequency component of the MSA recommendation and not verified full adherence, and it is labeled the MSA frequency threshold. For the primary analysis, meeting the MSA frequency threshold was defined as performing MSA ≥2 days/week; MSA frequency was additionally modeled as a three-level variable (0, 1, ≥2 days/week), with 0 days/week as the reference category, to compare 1 day/week and ≥2 days/week with no MSA.

#### 2.3.4. Covariates

Participants self-reported their age, sex, educational level, annual household income (in Saudi riyals, SAR), marital status, employment status, province of residence, height, and weight. Age was categorized into four groups (19–29, 30–39, 40–49, and ≥50 years); the 50–59 and 60–75 subgroups were combined owing to small cell counts that produced unstable estimates in stratified and interaction models. Body Mass Index (BMI) was calculated as weight in kilograms divided by height in meters squared (kg/m^2^) and categorized according to the World Health Organization (WHO) criteria as underweight (<18.5 kg/m^2^), normal weight (18.5–24.9 kg/m^2^), overweight (25.0–29.9 kg/m^2^), or obesity (≥30.0 kg/m^2^) [29]. Guideline thresholds and standardized definitions were used for the exposures and the outcome. The IPAQ-SF is a validated instrument, whereas LBP status and MSA were assessed with brief self-report questions.

### 2.4. Statistical Analysis

Participant characteristics were summarized using frequencies and percentages for categorical variables and means with standard deviations or medians with interquartile ranges for continuous variables as appropriate. The distribution of continuous variables was assessed through visual inspection of histograms and Q–Q plots, together with Shapiro–Wilk and Kolmogorov–Smirnov tests. Comparisons between participants with and without LBP were performed using the Mann–Whitney U test for continuous variables. For categorical variables, the Fisher exact test was used for 2 × 2 tables; for larger tables, the Pearson chi-square test was used, or the Fisher-Freeman-Halton exact test when more than 20% of cells had an expected count below 5. The test used for each comparison is marked beside its *p* value. Crude odds ratios (ORs) with 95% confidence intervals (CIs) were calculated for each PA and MSA category.

Multivariable binary logistic regression was used to examine the associations between PA/MSA and LBP status, with separate models for meeting the combined MVPA, moderate-intensity PA, vigorous-intensity PA, and MSA frequency thresholds. Age, sex, and BMI were prespecified as covariates based on their established associations with LBP and their potential relationships with PA and MSA. The main models were limited to these three covariates, as planned. Education, employment status, marital status, household income, and region were collected to describe the sample. They are plausible confounders, related to both activity and LBP, and were added in a sensitivity analysis described below. Covariates were not selected on the basis of univariate significance. All covariates were entered simultaneously, and age group and BMI category were coded identically in every model (reference categories: female, 19–29 years, normal weight). The results are presented as adjusted odds ratios (aORs) with 95% CIs. Interaction terms between sex and each threshold (sex × combined PA, sex × moderate PA, sex × vigorous PA, and sex × MSA) were evaluated in separate models to assess whether sex modified the PA/MSA-LBP associations. MSA frequency (0, 1, and ≥2 days/week, with 0 days/week as the reference) was additionally examined in a separate model with the same covariates. Because that model compares each category with no MSA, the direct contrast between ≥2 days/week and 1 day/week was also estimated by refitting the model with 1 day/week as the reference, and linear trend was tested with the three categories entered as an ordinal term. In line with the study objectives, two tests were primary: meeting the combined MVPA threshold and meeting the MSA frequency threshold. The moderate- and vigorous-intensity models and the three-level MSA frequency model were secondary; the frequency model is a second specification of the same exposure and not independent confirmation of the MSA result. The sex-interaction models, the continuous PA models, the direct contrast, the trend test, and the sensitivity analyses were exploratory. No adjustment was made for multiple comparisons: the two primary comparisons were set by the study objectives, and the other analyses are reported as secondary or exploratory. *p* values are therefore nominal. For reference, a Bonferroni threshold for the two primary tests would be *p <* 0.025.

Because dichotomizing PA by the guideline threshold may obscure associations across the full activity distribution, exploratory analyses examined PA as a continuous measure. The total combined moderate-equivalent PA, moderate-intensity PA, and vigorous-intensity minutes/week were positively skewed and were transformed using ln(x + 1), which retained observations with zero activity, and separate models were fitted for each log-transformed measure, adjusting for age, sex, and BMI. These models assume a monotonic association on the log scale and would not detect a U-shaped or other non-monotonic pattern.

Two sensitivity analyses were run. First, the models were refitted with additional adjustment for education (secondary or less, bachelor’s, master’s, doctorate), employment status, marital status (single, married, previously married), region (central, southern, other), and household income (<10,000; 10,000–29,000; 30,000–89,000; ≥90,000 SAR; prefer not to answer); categories with few participants were combined. Second, the 81 respondents with LBP lasting <3 months were returned to the sample and classified as not having chronic LBP (*N* = 530), and the main models were refitted. The results are given in Supplementary Table S1.

Before regression analyses, PA variables were cleaned according to the IPAQ-SF scoring rules [26]: reported session durations > 180 min were reduced to 180 min, as extreme single-session values were considered unreliable self-reports, and weekly sums and the moderate-equivalent PA variable were calculated from the reduced values. Multicollinearity was assessed using tolerance and variance inflation factor (VIF) statistics. Linearity of the logit for continuous BMI was assessed using the Box-Tidwell procedure, in which a BMI × ln(BMI) term is added to the model and a significant term indicates that the logit is not linear in BMI. It was decided before the final models were fitted that, if linearity was violated, BMI would be entered in the WHO categories instead of as a continuous variable, because these categories are the standard classification and allow comparison with other studies. No other specifications of BMI were evaluated. No missing data were present for the variables included in the primary analyses; therefore, no imputation was performed and every model used the full analytical sample (*N* = 449), apart from the sensitivity analysis that used all 530 respondents. All statistical tests were two-tailed, and statistical significance was set at *p <* 0.05. Statistical analyses were performed using IBM SPSS Statistics version 27.0.1 (IBM Corp., Armonk, NY, USA).

## 3. Results

### 3.1. Participant Characteristics

In total, 530 eligible individuals completed the survey, all of whom had passed the screening questions. No respondent reported LBP attributable to a specific pathology. Of these, 81 (15.3%) with LBP lasting <3 months were excluded under the prespecified outcome definition, yielding a final analytical sample of 449 participants. Of those included, 60.1% were female, 43.2% were aged 19–29 years, 48.1% were single, 46.1% were employed, and 40.5% were students. Participants were predominantly from the central (56.6%) and southern (34.3%) regions. Mean BMI was 26.6 ± 6.2 kg/m^2^; 37.2% were overweight and 20.9% were obese (Table 1).

### 3.2. Low Back Pain, Physical Activity, and Muscle-Strengthening Activity

Of the 449 participants, 239 (53.2%) were classified as having LBP lasting ≥3 months, and 210 (46.8%) as not having LBP. Among participants with LBP, the mean symptom duration was 46.95 ± 55.64 months. For PA, 70.6% and 65.3% of participants reported moderate- and vigorous-intensity activity, respectively, and 56.8% (255/449) met the combined MVPA threshold. MSA was reported by 42.3% of the participants, and 28.3% reported it on ≥2 days/week (Table 2).

### 3.3. Participant Characteristics According to LBP Status

The prevalence of LBP differed significantly across several characteristics. LBP was reported by 57.8% of females and 46.4% of males (*p* = 0.020), and its prevalence was 34.5% among participants aged 19–29 years and between 65.5% and 68.9% in each older age group (*p <* 0.001). Significant associations were also observed with marital status, education, household income, employment status, and province of residence (*p <* 0.001 for all except household income, *p* = 0.031). Participants with LBP had higher BMI than those without LBP (*p <* 0.001), and LBP prevalence differed across BMI categories (*p <* 0.001) (Table 3).

### 3.4. Associations Between Meeting PA/MSA Thresholds and LBP

Tests of regression assumptions showed a violation of the linearity-of-the-logit assumption for BMI as a continuous predictor (*p* = 0.048; Table 4); therefore, BMI Category (WHO classification) was used in all regression models. Tolerance values > 0.20 and VIF values < 5 for all predictors indicated no evidence of problematic multicollinearity among sex, age category, BMI category, and meeting the combined MVPA threshold (Table 5).

After adjusting for age, sex, and BMI, meeting the combined MVPA threshold was not significantly associated with LBP (aOR = 0.904; 95% CI, 0.599–1.364; *p* = 0.631) (Table 6). Similarly, no significant associations were observed for the moderate-intensity PA threshold (aOR = 0.813; 95% CI, 0.532–1.244; *p* = 0.340) (Table 7) or the vigorous-intensity PA threshold (aOR = 0.935; 95% CI, 0.618–1.416; *p* = 0.752) (Table 8). Meeting the MSA frequency threshold was associated with lower odds of LBP (aOR = 0.606; 95% CI, 0.385–0.954; *p* = 0.031) (Table 9); this result is nominally significant and statistically borderline. The MSA frequency model re-specifies the same exposure in three levels and is not an independent test. In it, compared with no MSA, ≥2 days/week was associated with lower odds of LBP after adjustment for age, sex, and BMI (aOR = 0.600; 95% CI, 0.376–0.959; *p* = 0.033), while 1 day/week was not significantly associated with LBP (aOR = 0.949; 95% CI, 0.515–1.748; *p* = 0.866) (Table 10). Both estimates compare a category with no MSA and do not show that the two categories differ from each other. In the direct contrast, the odds of LBP at ≥2 days/week were not significantly different from those at 1 day/week (aOR = 0.632; 95% CI, 0.323–1.239; *p* = 0.182). The test for linear trend across the three categories was nominally significant (aOR per category = 0.783; 95% CI, 0.621–0.988; *p* = 0.039), whereas the overall 2-df test of the frequency variable was not (*p* = 0.095). These data do not identify a frequency threshold.

Across all models, older age was consistently associated with higher odds of LBP, while male sex was consistently associated with lower odds of LBP. BMI, entered as a categorical variable, was not significantly associated with LBP after adjusting for the other model variables, although obese participants showed a non-significant trend toward higher odds relative to those with normal weight (Table 6, Table 7, Table 8, Table 9 and Table 10).

### 3.5. Interaction Between Sex and PA/MSA

No statistically significant evidence of effect modification by sex was detected for the associations between meeting the PA/MSA thresholds and LBP. Interaction terms were not statistically significant for combined PA (aOR = 1.123; 95% CI, 0.491–2.572; *p* = 0.783), moderate-intensity PA (aOR = 0.730; 95% CI, 0.303–1.757; *p* = 0.482), vigorous-intensity PA (aOR = 1.155; 95% CI, 0.501–2.663; *p* = 0.735), or MSA (aOR = 1.364; 95% CI, 0.546–3.407; *p* = 0.507) (Table 11, Table 12, Table 13 and Table 14). The confidence intervals were wide, so a meaningful difference between women and men cannot be excluded.

### 3.6. Exploratory Analysis Using Continuous PA Measures

Exploratory analyses using log-transformed continuous PA measures also showed no significant associations. After adjusting for age, sex, and BMI, none of the total combined PA (aOR = 0.985; 95% CI, 0.913–1.062; *p* = 0.686), moderate-intensity PA (aOR = 0.951; 95% CI, 0.876–1.033; *p* = 0.238), or vigorous-intensity PA (aOR = 1.004; 95% CI, 0.926–1.089; *p* = 0.919) was significantly associated with LBP (Table 15, Table 16 and Table 17).

### 3.7. Crude and Adjusted Associations by Exposure Category

Table 18 gives the number and proportion of participants with LBP in each PA and MSA category, with crude and adjusted odds ratios. LBP was reported by 51.0% of participants who met the combined MVPA threshold and 56.2% of those who did not, and by 57.9%, 52.4%, and 44.1% of participants reporting MSA on 0, 1, and ≥2 days/week. Adjustment for age, sex, and BMI moved the PA estimates toward 1 (for example, vigorous-intensity PA from a crude OR of 0.755 to an aOR of 0.935) and changed the estimate for MSA on ≥2 days/week little (crude OR 0.573; aOR 0.600). All models in Table 18 used the full analytical sample (*N* = 449); no participant had missing data on any model variable.

### 3.8. Sensitivity Analyses

Two sensitivity analyses are reported in Supplementary Table S1. Additional adjustment for education, employment status, marital status, region, and household income changed the estimates little: meeting the MSA frequency threshold remained associated with lower odds of LBP (aOR = 0.567; 95% CI, 0.355–0.905; *p* = 0.018), the PA estimates remained non-significant, and the contrast between ≥2 days/week and 1 day/week remained non-significant (aOR = 0.589; 95% CI, 0.292–1.187; *p* = 0.139). When the 81 respondents with LBP lasting <3 months were kept in the sample as non-cases (*N* = 530; 239 with chronic LBP and 291 without), meeting the MSA frequency threshold was still associated with lower odds of chronic LBP (aOR = 0.630; 95% CI, 0.415–0.957; *p* = 0.030), and the estimates for the combined MVPA (aOR = 0.801; 95% CI, 0.549–1.167), moderate-intensity PA (aOR = 0.753; 95% CI, 0.511–1.108), and vigorous-intensity PA thresholds (aOR = 1.031; 95% CI, 0.705–1.507) remained non-significant.

## 4. Discussion

### 4.1. Principal Findings

This study found no clear evidence that meeting the international thresholds for combined, moderate-intensity, or vigorous-intensity physical activity (PA) was associated with chronic low back pain (LBP) among Saudi adults, while muscle-strengthening activity (MSA) on ≥2 days/week was associated with lower odds of LBP than MSA on fewer than 2 days/week. The estimate for 1 day/week was close to 1 and imprecise, and it did not differ significantly from the estimate for ≥2 days/week, so the data do not establish a frequency threshold. The MSA estimate is statistically borderline (95% CI, 0.385–0.954; *p* = 0.031). Age and sex were independently associated with LBP; the unadjusted association with BMI did not survive adjustment. These results are better read as two separate findings than as a single “PA doesn’t matter” result. None of the aerobic PA specifications examined was significantly associated with LBP, whereas MSA at the recommended frequency was. Whether the PA and MSA associations differ from each other was not tested.

### 4.2. Physical Activity and Low Back Pain

The non-significant PA result fits an already inconsistent literature. Shiri and Falah-Hassani’s review of 36 prospective cohorts found leisure-time PA to be modestly protective against LBP overall, with less consistent evidence for vigorous activity specifically [10]. An earlier cross-sectional study by Heneweer et al. went further and raised the possibility that the PA-LBP relationship is U-shaped rather than linear [12], a pattern that contradicts “more PA is better” and that a guideline-threshold analysis like this one is poorly suited to detect. A later meta-analysis of nearly 96,000 adults similarly found moderate PA associated with modestly lower LBP risk, but no additional benefit at higher volumes [11]. A systematic review by Heneweer et al. found that occupational physical loading was more consistently associated with LBP than leisure-time activity or exercise [30], pointing toward the activity domain, not volume, as the more relevant variable. A guideline expressed in minutes/week treats occupational lifting and recreational walking identically, with no obvious reason to expect equivalent spinal consequences.

This may partly explain why these findings diverge from those of a study in middle-aged and older Chinese adults, where sufficient PA was associated with lower odds of chronic LBP [25]. That study scored PA with the IPAQ total, which counts walking, so its exposure differs from the one used here. Its sample was also restricted to adults aged ≥45 years, and occupational structure and activity culture plausibly differ between settings in which a shared minutes-per-week threshold does not need to produce a shared association. Power is a further concern. The sample size assessment used the proportions reported by the same Chinese study [25], where sufficient PA was linked to a considerably larger reduction in LBP odds than typically observed in the literature. If the true association among Saudi adults is smaller, this study may have lacked the power to detect it. Walking was also not counted toward the PA thresholds (Section 2.3.2), so some adults who are active mainly through brisk walking were classified as not meeting them. Misclassification of this kind could dilute a true association. The confidence intervals (for example, 0.599 to 1.364 for the combined MVPA threshold) are compatible with moderately lower odds, no association, or somewhat higher odds. The non-significant PA result is therefore inconclusive and does not show that no association exists. The same applies to the sex interaction tests, for which the sample size was not calculated.

The PA estimates were non-significant whether PA was treated as a guideline threshold, an intensity category, or a continuous log-transformed variable. That consistency covers only the specifications examined. The log-transformed models assume a monotonic association and would not detect a U-shaped or other non-monotonic pattern, so the result cannot be claimed to be independent of how PA was specified. The IPAQ-SF also measures frequency and duration, not movement quality, load, or lifetime exposure, and the cross-sectional design cannot separate “PA doesn’t affect LBP” from “people in pain move differently, in ways that cancel out any true association.”

### 4.3. Muscle-Strengthening Activity and Low Back Pain

LBP is increasingly understood as a biopsychosocial condition shaped by interacting mechanical, behavioral, and psychosocial contributors rather than any single exposure [3,31], and the different patterns for PA and MSA observed here would fit that framing better than a model in which “activity” is one undifferentiated exposure, although the difference between them was not tested. Exercise-based interventions, including resistance and stabilization training, have shown benefits for existing chronic LBP in a systematic review [13], and a meta-analysis of controlled trials found that exercise programs lowered the risk of new LBP episodes [14]. Treating existing chronic LBP and preventing new episodes are both different questions from the one this study asks, whether habitual MSA is associated with lower odds of chronic LBP in the general population, but they lend plausibility to a mechanism distinct from aerobic conditioning.

Compared with no MSA, the odds of LBP were lower at ≥2 days per week, a nominally significant and statistically borderline result, and the estimate at 1 day per week was not significant. This pattern is not evidence of a threshold. Only 63 participants reported 1 day/week, the confidence interval for that category was wide (0.515 to 1.748), and the direct contrast between ≥2 days/week and 1 day/week was not significant (aOR = 0.632; 95% CI, 0.323–1.239). The trend across the three categories was nominally significant (*p* = 0.039), which would fit a graded association as well as a threshold, and this study cannot distinguish the two. Dose–response work in exercise-therapy trials for existing LBP has found some support for frequency-sensitive effects: a meta-regression of stabilization-exercise trials found low-quality evidence that training 3–5 times weekly produced the largest treatment effect in an inverted U-shaped pattern [32]. That literature concerns treatment dose in symptomatic patients, not habitual exposure in the general population, and the frequency finding itself was graded low quality. It cannot explain this study’s result directly, but it shows that frequency-sensitive effects of spine-relevant exercise have been reported, if with uncertainty.

These results are broadly in line with a US study of National Health and Nutrition Examination Survey (NHANES) participants that reported lower odds of LBP among adults doing MSA on ≥2 days/week than among those doing none [15]. Its estimates were stratified by sex, and after further adjustment for smoking the association remained significant in women (*p* = 0.03) but not in men (*p* = 0.21). That result does not by itself show a sex difference, and smoking was not measured in the present study. Both studies are cross-sectional, and neither can establish that MSA precedes reduced LBP rather than following from it; pain-free individuals may simply find it easier to sustain a twice-weekly strengthening routine, and adults with chronic LBP may have cut back or stopped strengthening activity because of their pain. Either would produce the association seen here without MSA having any influence on LBP. To the author’s knowledge, no earlier study has examined the MSA frequency threshold and chronic LBP in Saudi adults.

The MSA classification also has limits. A frequency-based classification says nothing about exercise type, intensity, load, sets and repetitions, session duration, technique, supervision, training history, or muscle groups trained; two people who both report ≥2 days/week could have quite different physiological exposures. This is why the exposure is described as the frequency component of the recommendation and not as full guideline adherence. The finding indicates that frequency of engagement is associated with LBP; it does not show that frequency is the active ingredient. The study did not test a resistance-training program, so it says nothing about the effectiveness of prescribed strengthening exercise.

### 4.4. Age, Sex, and BMI

Older age and female sex were independently associated with higher odds of LBP across all models, consistent with Saudi evidence: a published meta-analysis found higher LBP prevalence in women than in men [6], and in the author’s unpublished review older age and female sex were each associated with higher pooled odds of LBP [7]. Sex disparities have been linked to a combination of biomechanical, hormonal, and psychosocial factors [33,34], including a documented increase in LBP prevalence among women following menopause [35]. Both associations held after adjustment for PA and MSA in every model, so activity alone does not explain them.

The association of BMI with LBP did not survive adjustment for age, sex, and PA/MSA, although it was significant unadjusted. This does not mean that BMI is irrelevant to LBP. A meta-analysis reported that obesity was associated with LBP, including chronic LBP, and that the association held in studies that controlled for confounders [36], so the present result differs from that evidence. The adjusted estimates for obesity here (aOR 1.39 to 1.44 across models) were similar in size to those pooled estimates but imprecise, with confidence intervals that included 1, so this study may have lacked the precision to detect an independent BMI association. The unadjusted BMI association may also have been partly confounded by age and sex. Elevated BMI may still contribute to LBP risk through mechanisms established elsewhere in obesity-LBP literature, including increased spinal loading [36].

### 4.5. Implications

These findings do not support changes to clinical or preventive recommendations, and they cannot support prescribing MSA to prevent LBP. What they support is further study of whether aerobic PA and MSA relate to LBP differently, which means measuring and analyzing the two separately when LBP is the outcome of interest. General aerobic PA guidance remains important for reasons established elsewhere: cardiovascular, metabolic, and broader chronic disease prevention [8,9]. In this sample there was no clear evidence that it was associated with chronic LBP, whereas MSA on ≥2 days/week was associated with lower odds than no MSA. The data do not show that one weekly session differs from two or more, and they give no basis for advice about “some” versus “sufficient” MSA. The design is cross-sectional and the sample was recruited online rather than in clinics, so the findings do not show that promoting MSA would prevent LBP. A prospective cohort study in clinical and other community populations is the appropriate next step, followed by a randomized trial if the association holds. The question matters in the Saudi context, where physical inactivity remains prevalent [16] and LBP represents a substantial population burden [6,7]. The current WHO guidance on chronic primary LBP management already emphasizes multidisciplinary approaches over single-modality intervention [19]. If MSA does prove useful for prevention, it would be one component of a broader strategy that also addresses occupational loading, ergonomics, and psychosocial contributors.

### 4.6. Strengths and Limitations

This study has several strengths. To the author’s knowledge, no Saudi study of adults has combined separate comparisons for the non-walking aerobic PA threshold and the MSA frequency threshold, rather than crude activity levels, with a self-reported chronic LBP outcome. The use of several analytic approaches (guideline-threshold, intensity-stratified, continuous, and frequency-based models, alongside sex-interaction testing and sensitivity analyses) showed how far the PA and MSA results depended on model specification, within the specifications examined; it did not rule out non-monotonic associations. The use of the IPAQ-SF, a validated self-report instrument, and internationally standardized guideline thresholds also supports comparability with future studies in this and other populations.

This study has several limitations that warrant consideration. The cross-sectional design is the central constraint on every result, applying equally to the non-significant PA finding and the MSA finding; reverse causation is plausible for both. PA and MSA were self-reported, which risks recall and social-desirability bias despite the IPAQ-SF’s established validity, and this matters for the MSA frequency categories in particular. MSA was assessed with two brief questions over a 30-day recall period that captured frequency only, not intensity or the muscle groups worked, PA with the IPAQ-SF over 7 days, and LBP status with two self-report questions and no measure of pain intensity or disability. The outcome is therefore the presence of self-reported chronic LBP, and the findings say nothing about pain severity or function. The recall windows also differed: 7 days for PA, 30 days for MSA, and 3 months or longer for LBP. For participants with chronic LBP, both recall windows fell within the period of pain, and the survey did not ask whether activity had changed after the pain began. It therefore cannot be determined whether the activity reported reflects habitual activity before the pain or a change afterwards, which adds to exposure misclassification and to the possibility of reverse causation. Objective measurement would sharpen these comparisons. Chronic LBP was defined by duration only, without the pain-frequency criterion of the NIH Task Force definition, which limits comparability with studies that applied that definition. Walking was not counted toward the PA thresholds, so adults active mainly through brisk walking may have been misclassified as not meeting them. Eligibility relied on self-report in an anonymous online survey. Because LBP status was assessed only at the time of the survey, the comparison group may include people with a history of LBP who were asymptomatic when surveyed, including some who may have changed their activity as part of recovery. These participants are not misclassified, because the outcome is current chronic LBP, but their presence could affect the associations. The direction of any such bias depends on recovery, changes in activity, and selection, and cannot be determined from these data. Excluding the 81 respondents (15.3%) with LBP lasting <3 months made the comparison one between chronic LBP and no current LBP. It also produced a selected analytical sample, and the associations could differ if people with acute or subacute LBP were retained. In a sensitivity analysis that kept these respondents as non-cases (*N* = 530), the MSA estimate was similar (aOR = 0.630; 95% CI, 0.415–0.957) and the PA estimates remained non-significant (Table S1). Neither PA nor MSA measurements captured domain, exercise type, or lifetime loading. The IPAQ-SF does not separate occupational from leisure activity: one participant can meet a PA threshold through physically demanding work that loads the spine, and another can report little recreational activity but have a heavy job. Guideline-based PA is therefore not a measure of the mechanical exposure relevant to LBP, which is a source of both exposure misclassification and residual confounding. The main models adjusted for age, sex, and BMI only. Adding education, employment status, marital status, region, and household income in a sensitivity analysis did not materially change the estimates (Table S1), but smoking, psychological distress, comorbidities, occupational loading and posture, sleep, and previous LBP were not measured, and sitting time, recorded as free text, was not analyzed, so residual confounding cannot be excluded for either result. Recruitment through WhatsApp groups and university email lists skewed the sample toward younger adults (43.2% aged 19–29 years), students (40.5%), and the central and southern regions (90.9%), and only people who chose to respond were included. The sample is not representative of the Saudi adult population. The results describe this self-selected online sample and cannot be generalized to the wider Saudi population, older adults, patients seeking care for LBP, occupational groups, or other cultural settings, where the associations could differ. The sample size assessment was based on a PA comparison using proportions from a study of adults aged ≥45 years, not for MSA or the sex-interaction analyses, so power for those analyses is unknown, and the nominal significance of the MSA result is not evidence that the study was adequately powered for it. This study’s analyses were not formally preregistered. The guideline-threshold models, the three-level MSA frequency model, and the sex-interaction models were planned before data analysis; the direct contrast, the trend test, and the sensitivity analyses were added after the main analyses. Two tests were primary (the combined MVPA threshold and the MSA frequency threshold); the intensity-specific and MSA frequency models were secondary, and the interaction, continuous, contrast, trend, and sensitivity analyses were exploratory and should be considered hypothesis-generating. No correction was made for multiple comparisons, so all *p* values are nominal and the chance of at least one type I error across the full set of tests is above 5%. The MSA association was the one hypothesized to be strongest, but its *p* value (0.031) is close to the conventional threshold and would not meet a Bonferroni threshold for the two primary tests (*p <* 0.025), and the upper confidence limit was close to 1 (0.954). The frequency model (≥2 days/week, *p* = 0.033) is a second specification of the same exposure, not independent confirmation. The MSA finding is therefore preliminary and needs replication.

## 5. Conclusions

In this cross-sectional sample of Saudi adults, MSA on ≥2 days/week was associated with lower odds of chronic LBP (aOR = 0.606; 95% CI, 0.385–0.954), and no statistically significant association was detected for the aerobic PA thresholds. The MSA estimate is statistically borderline, and both exposure and outcome were self-reported. The data do not establish a frequency threshold for MSA or a difference between the PA and MSA associations. The design cannot show whether strengthening activity lowers the risk of LBP or whether adults without LBP find it easier to keep up a strengthening routine. The finding is preliminary and comes from a self-selected online sample, so it should be tested in longitudinal studies with validated MSA and LBP measures before it informs prevention advice.

## Figures and Tables

**Table 1:** Sociodemographic and Anthropometric Characteristics of the Study Participants (*N* = 449).

		*N*	%
Gender	Female	270	60.1%
	Male	179	39.9%
Age	19–29	194	43.2%
	30–39	87	19.4%
	40–49	103	22.9%
	≥50	65	14.5%
Marital Status	Single	216	48.1%
	Married	212	47.2%
	Divorced	11	2.4%
	Separated	7	1.6%
	Widow	3	0.7%
Education Level	Less than a high school diploma	5	1.1%
	General Secondary Certificate	142	31.6%
	Bachelor’s degree	184	41.0%
	Master’s degree	78	17.4%
	PhD	40	8.9%
Total Family Annual Income (SAR)	<10,000	75	16.7%
	10,000–29,000	130	29.0%
	30,000–49,000	33	7.3%
	50,000–69,000	20	4.5%
	70,000–89,000	8	1.8%
	90,000–149,000	15	3.3%
	>150,000	47	10.5%
	Prefer not to answer	121	26.9%
Current Employment Status	Employed	207	46.1%
	Not Employed	60	13.4%
	Student	182	40.5%
Province of Residence	Central	254	56.6%
	East	19	4.2%
	North	5	1.1%
	South	154	34.3%
	West	17	3.8%
BMI (kg/m^2^)	Mean ± SD	26.6 ± 6.2	
BMI Category	Underweight (<18.5 kg/m^2^)	18	4.0%
	Normal weight (18.5–24.9 kg/m^2^)	170	37.9%
	Overweight (25.0–29.9 kg/m^2^)	167	37.2%
	Obese (≥30.0 kg/m^2^)	94	20.9%

**Table 2:** Low Back Pain Characteristics, Physical Activity, and Muscle-Strengthening Activity Among the Study Participants (*N* = 449).

		*N*	%
LBP Status	No	210	46.8%
	Yes	239	53.2%
LBP Duration (Months)	Mean ± SD	46.95 ± 55.64	
Vigorous PA	No	156	34.7%
	Yes	293	65.3%
Days per Week of Vigorous PA (*N* = 293)	1	67	22.9%
	2	71	24.2%
	3	66	22.5%
	4	37	12.6%
	5	30	10.2%
	6	9	3.1%
	7	13	4.4%
Vigorous PA Duration per Session (Minutes) (*N* = 293) *	Mean ± SD	43.14 ± 52.6	
Total Weekly Vigorous PA (Minutes) (*N* = 293) *	Mean ± SD	140.8 ± 211.4	
Meets Vigorous PA Threshold (≥75 min/week)	No	258	57.5%
	Yes	191	42.5%
Moderate PA	No	132	29.4%
	Yes	317	70.6%
Days per Week of Moderate PA (*N* = 317)	1	57	18.0%
	2	73	23.0%
	3	66	20.8%
	4	46	14.5%
	5	45	14.2%
	6	13	4.1%
	7	17	5.4%
Moderate PA Duration per Session (Minutes) (*N* = 317) *	Mean ± SD	47.5 ± 54	
Total Weekly Moderate PA (Minutes) (*N* = 317) *	Mean ± SD	170.7 ± 248.6	
Meets Moderate PA Threshold (≥150 min/week)	No	291	64.8%
	Yes	158	35.2%
Meets Combined MVPA Threshold (≥150 min/week)	No	194	43.2%
	Yes	255	56.8%
Walking 10 Minutes or More (Days/Week)	0	41	9.1%
	1	34	7.6%
	2	66	14.7%
	3	71	15.8%
	4	44	9.8%
	5	56	12.5%
	6	29	6.5%
	7	108	24.1%
MSA Participation	No	259	57.7%
	Yes	190	42.3%
MSA (Days/Week) (*N* = 190)	1 day a week	63	33.2%
	≥2 days per week	127	66.8%
Meets MSA Frequency Threshold (≥2 Days/Week)	No	322	71.7%
	Yes	127	28.3%

Note. LBP cases were defined as participants reporting LBP lasting ≥3 months. * Sample sizes for days/week, duration-per-session, and weekly-total variables reflect only participants reporting engagement in that PA subcategory; participants reporting no activity were assigned zero minutes/week and are included in full (*N* = 449) in the threshold rows above.

**Table 3:** Comparison of Sociodemographic and Anthropometric Characteristics According to Low Back Pain Status.

		Low Back Pain Status		
		No	Yes	*p* Value
		*N* (%)	*N* (%)	
Gender	Female	114 (42.2%)	156 (57.8%)	0.020 *^,F^
	Male	96 (53.6%)	83 (46.4%)	
Age	19–29	127 (65.5%)	67 (34.5%)	<0.001 *^,C^
	30–39	30 (34.5%)	57 (65.5%)	
	40–49	32 (31.1%)	71 (68.9%)	
	≥50	21 (32.3%)	44 (67.7%)	
Marital Status	Divorced	2 (18.2%)	9 (81.8%)	<0.001 *^,H^
	Married	73 (34.4%)	139 (65.6%)	
	Separated	3 (42.9%)	4 (57.1%)	
	Single	132 (61.1%)	84 (38.9%)	
	Widow	0 (0.0%)	3 (100.0%)	
Education Level	Less than a high school	1 (20.0%)	4 (80.0%)	<0.001 *^,C^
	General Secondary Certificate	85 (59.9%)	57 (40.1%)	
	Bachelor’s degree	83 (45.1%)	101 (54.9%)	
	Master’s degree	33 (42.3%)	45 (57.7%)	
	PhD	8 (20.0%)	32 (80.0%)	
Total Family Annual Income (SAR)	<10,000	43 (57.3%)	32 (42.7%)	0.031 *^,C^
	10,000–29,000	61 (46.9%)	69 (53.1%)	
	30,000–49,000	7 (21.2%)	26 (78.8%)	
	50,000–69,000	9 (45.0%)	11 (55.0%)	
	70,000–89,000	6 (75.0%)	2 (25.0%)	
	90,000–149,000	7 (46.7%)	8 (53.3%)	
	>150,000	19 (40.4%)	28 (59.6%)	
	Prefer not to answer	58 (47.9%)	63 (52.1%)	
Current Employment Status	Employed	76 (36.7%)	131 (63.3%)	<0.001 *^,C^
	Not Employed	19 (31.7%)	41 (68.3%)	
	Student	115 (63.2%)	67 (36.8%)	
Province of Residence	Central	102 (40.2%)	152 (59.8%)	<0.001 *^,C^
	East	4 (21.1%)	15 (78.9%)	
	North	4 (80.0%)	1 (20.0%)	
	South	95 (61.7%)	59 (38.3%)	
	West	5 (29.4%)	12 (70.6%)	
BMI (kg/m^2^)	Mean ± SD	25.4 ± 6.1	27.6 ± 6.0	<0.001 *^,U^
BMI Category	Underweight (<18.5 kg/m^2^)	15 (83.3%)	3 (16.7%)	<0.001 *^,C^
	Normal weight (18.5–24.9 kg/m^2^)	91 (53.5%)	79 (46.5%)	
	Overweight (25.0–29.9 kg/m^2^)	73 (43.7%)	94 (56.3%)	
	Obese (≥30.0 kg/m^2^)	31 (33.0%)	63 (67.0%)	

^U^ Independent Sample Mann–Whitney U test; ^C^ Pearson Chi-Square test (larger tables); ^F^ Fisher’s Exact test (2 × 2 tables); ^H^ Fisher-Freeman-Halton Exact test (larger tables with more than 20% of expected counts below 5); * *p <* 0.05, Significant.

**Table 4:** Assessment of Logistic Regression Assumptions: Linearity of the Logit.

	B	S.E.	Wald	df	aOR	95% CI		*p* Value
						Lower	Upper	
BMI × Ln (BMI)	−0.141	0.072	3.905	1	0.868	0.755	0.999	0.048 *

Predictors: Gender, Age, Body Mass Index (BMI) (kg/m^2^), BMI × Ln (BMI), Meets Combined MVPA Threshold. Outcome: Low Back Pain; * *p <* 0.05, Significant.

**Table 5:** Assessment of Logistic Regression Assumptions: Multicollinearity (Tolerance/VIF).

Variable	Tolerance	VIF
Gender (Male vs. Female)	0.936	1.068
Age Categories	0.780	1.282
BMI Categories	0.834	1.200
Meets Combined MVPA Threshold	0.982	1.019

**Table 6:** Multivariable Logistic Regression Analysis of Factors Associated with Low Back Pain According to Meeting the Combined Moderate-to-Vigorous Physical Activity Threshold.

		B	S.E.	Wald	df	aOR	95% CI		*p* Value
							Lower	Upper	
Gender	Female	Ref	Ref	Ref	Ref	Ref	Ref	Ref	Ref
	Male	−0.932	0.227	16.786	1	0.394	0.252	0.615	<0.001 *
Age	19–29	Ref	Ref	Ref	Ref	Ref	Ref	Ref	Ref
	30–39	1.237	0.290	18.209	1	3.446	1.952	6.083	<0.001 *
	40–49	1.540	0.301	26.186	1	4.667	2.587	8.419	<0.001 *
	≥50	1.587	0.348	20.738	1	4.887	2.469	9.675	<0.001 *
BMI Category	Normal (18.5–24.9)	Ref	Ref	Ref	Ref	Ref	Ref	Ref	Ref
	Underweight (<18.5)	−1.060	0.665	2.543	1	0.346	0.094	1.275	0.111
	Overweight (25.0–29.9)	0.042	0.250	0.028	1	1.043	0.639	1.703	0.866
	Obese (≥30.0)	0.352	0.301	1.369	1	1.423	0.788	2.568	0.242
Meets Combined MVPA Threshold	No	Ref	Ref	Ref	Ref	Ref	Ref	Ref	Ref
	Yes	−0.101	0.210	0.231	1	0.904	0.599	1.364	0.631

Predictors: Gender, Age, Body Mass Index (BMI), Meets Combined MVPA Threshold (≥150 moderate-equivalent min/week, walking not counted). Outcome: Low Back Pain; * *p <* 0.05, Significant.

**Table 7:** Multivariable Logistic Regression Analysis of Factors Associated with Low Back Pain According to Meeting the Moderate-Intensity Physical Activity Threshold.

		B	S.E.	Wald	df	aOR	95% CI		*p* Value
							Lower	Upper	
Gender	Female	Ref	Ref	Ref	Ref	Ref	Ref	Ref	Ref
	Male	−0.937	0.228	16.944	1	0.392	0.251	0.612	<0.001 *
Age	19–29	Ref	Ref	Ref	Ref	Ref	Ref	Ref	Ref
	30–39	1.230	0.290	17.949	1	3.421	1.937	6.044	<0.001 *
	40–49	1.523	0.301	25.600	1	4.584	2.542	8.268	<0.001 *
	≥50	1.582	0.347	20.764	1	4.865	2.464	9.608	<0.001 *
BMI Category	Normal (18.5–24.9)	Ref	Ref	Ref	Ref	Ref	Ref	Ref	Ref
	Underweight (<18.5)	−1.065	0.665	2.567	1	0.345	0.094	1.268	0.109
	Overweight (25.0–29.9)	0.038	0.250	0.024	1	1.039	0.637	1.696	0.878
	Obese (≥30.0)	0.355	0.302	1.386	1	1.426	0.790	2.576	0.239
Meets Moderate PA Threshold	No	Ref	Ref	Ref	Ref	Ref	Ref	Ref	Ref
	Yes	−0.207	0.217	0.912	1	0.813	0.532	1.244	0.340

Predictors: Gender, Age, Body Mass Index (BMI), Meets Moderate PA Threshold (≥150 min/week). Outcome: Low Back Pain; * *p <* 0.05, Significant.

**Table 8:** Multivariable Logistic Regression Analysis Examining the Association Between Meeting the Vigorous-Intensity Physical Activity Threshold and Low Back Pain.

		B	S.E.	Wald	df	aOR	95% CI		*p* Value
							Lower	Upper	
Gender	Female	Ref	Ref	Ref	Ref	Ref	Ref	Ref	Ref
	Male	−0.930	0.227	16.726	1	0.395	0.253	0.616	<0.001 *
Age	19–29	Ref	Ref	Ref	Ref	Ref	Ref	Ref	Ref
	30–39	1.239	0.290	18.202	1	3.451	1.953	6.096	<0.001 *
	40–49	1.541	0.302	25.964	1	4.671	2.582	8.450	<0.001 *
	≥50	1.586	0.351	20.376	1	4.886	2.454	9.729	<0.001 *
BMI Category	Normal (18.5–24.9)	Ref	Ref	Ref	Ref	Ref	Ref	Ref	Ref
	Underweight (<18.5)	−1.044	0.664	2.475	1	0.352	0.096	1.293	0.116
	Overweight (25.0–29.9)	0.044	0.251	0.030	1	1.044	0.639	1.707	0.862
	Obese (≥30.0)	0.354	0.301	1.377	1	1.424	0.789	2.570	0.241
Meets Vigorous PA Threshold	No	Ref	Ref	Ref	Ref	Ref	Ref	Ref	Ref
	Yes	−0.067	0.212	0.100	1	0.935	0.618	1.416	0.752

Predictors: Gender, Age, Body Mass Index (BMI), Meets Vigorous PA Threshold (≥75 min/week). Outcome: Low Back Pain; * *p <* 0.05, Significant.

**Table 9:** Multivariable Logistic Regression Analysis of Factors Associated with Low Back Pain According to Meeting the Muscle-Strengthening Activity Frequency Threshold.

		B	S.E.	Wald	df	aOR	95% CI		*p* Value
							Lower	Upper	
Gender	Female	Ref	Ref	Ref	Ref	Ref	Ref	Ref	Ref
	Male	−0.902	0.229	15.510	1	0.406	0.259	0.636	<0.001 *
Age	19–29	Ref	Ref	Ref	Ref	Ref	Ref	Ref	Ref
	30–39	1.287	0.292	19.425	1	3.623	2.044	6.423	<0.001 *
	40–49	1.511	0.302	25.046	1	4.532	2.508	8.190	<0.001 *
	≥50	1.580	0.347	20.722	1	4.856	2.459	9.590	<0.001 *
BMI Category	Normal (18.5–24.9)	Ref	Ref	Ref	Ref	Ref	Ref	Ref	Ref
	Underweight (<18.5)	−1.176	0.667	3.107	1	0.308	0.083	1.141	0.078
	Overweight (25.0–29.9)	0.036	0.251	0.020	1	1.036	0.633	1.695	0.887
	Obese (≥30.0)	0.329	0.304	1.174	1	1.389	0.766	2.519	0.278
Meets MSA Frequency Threshold	No	Ref	Ref	Ref	Ref	Ref	Ref	Ref	Ref
	Yes	−0.501	0.232	4.673	1	0.606	0.385	0.954	0.031 *

Predictors: Gender, Age, Body Mass Index (BMI), Meets Muscle-Strengthening Activity (MSA) Frequency Threshold (≥2 Days/Week). Outcome: Low Back Pain; * *p <* 0.05, Significant.

**Table 10:** Multivariable Logistic Regression Analysis of Factors Associated with Low Back Pain According to Muscle Strengthening Activity Frequency.

		B	S.E.	Wald	df	aOR	95% CI		*p* Value
							Lower	Upper	
Gender	Female	Ref	Ref	Ref	Ref	Ref	Ref	Ref	Ref
	Male	−0.901	0.229	15.479	1	0.406	0.259	0.636	<0.001 *
Age	19–29	Ref	Ref	Ref	Ref	Ref	Ref	Ref	Ref
	30–39	1.285	0.292	19.334	1	3.616	2.039	6.413	<0.001 *
	40–49	1.507	0.303	24.793	1	4.515	2.494	8.171	<0.001 *
	≥50	1.581	0.347	20.736	1	4.861	2.461	9.600	<0.001 *
BMI Category	Normal (18.5–24.9)	Ref	Ref	Ref	Ref	Ref	Ref	Ref	Ref
	Underweight (<18.5)	−1.167	0.669	3.041	1	0.311	0.084	1.156	0.081
	Overweight (25.0–29.9)	0.037	0.251	0.022	1	1.038	0.634	1.699	0.883
	Obese (≥30.0)	0.331	0.304	1.185	1	1.392	0.768	2.523	0.276
MSA Frequency	0 days/week	Ref	Ref	Ref	Ref	Ref	Ref	Ref	Ref
	1 day/week	−0.052	0.312	0.028	1	0.949	0.515	1.748	0.866
	≥2 days/week	−0.511	0.239	4.567	1	0.600	0.376	0.959	0.033 *

Predictors: Gender, Age, Body Mass Index (BMI), Muscle Strengthening Activity (MSA) Frequency. Outcome: Low Back Pain; * *p <* 0.05, Significant.

**Table 11:** Multivariable Logistic Regression Analysis Assessing the Interaction Between Sex and Meeting the Combined Moderate-to-Vigorous Physical Activity Threshold in Relation to Low Back Pain.

		B	S.E.	Wald	df	aOR	95% CI		*p* Value
							Lower	Upper	
Gender	Female	Ref	Ref	Ref	Ref	Ref	Ref	Ref	Ref
	Male	−0.998	0.331	9.069	1	0.369	0.193	0.706	0.003 *
Age	19–29	Ref	Ref	Ref	Ref	Ref	Ref	Ref	Ref
	30–39	1.236	0.290	18.187	1	3.442	1.950	6.076	<0.001 *
	40–49	1.539	0.301	26.126	1	4.659	2.582	8.404	<0.001 *
	≥50	1.586	0.348	20.710	1	4.884	2.467	9.669	<0.001 *
BMI Category	Normal (18.5–24.9)	Ref	Ref	Ref	Ref	Ref	Ref	Ref	Ref
	Underweight (<18.5)	−1.064	0.665	2.557	1	0.345	0.094	1.271	0.110
	Overweight (25.0–29.9)	0.046	0.251	0.034	1	1.047	0.641	1.711	0.854
	Obese (≥30.0)	0.355	0.301	1.384	1	1.426	0.790	2.573	0.239
Meets Combined MVPA Threshold	No	Ref	Ref	Ref	Ref	Ref	Ref	Ref	Ref
	Yes	−0.150	0.276	0.296	1	0.861	0.501	1.478	0.587
Interaction: Gender × Meeting Combined MVPA Threshold	Female × No	Ref	Ref	Ref	Ref	Ref	Ref	Ref	Ref
	Male × Yes	0.116	0.423	0.076	1	1.123	0.491	2.572	0.783

Predictors: Gender, Age, Body Mass Index (BMI), Meets Combined MVPA Threshold (≥150 moderate-equivalent min/week), Interaction: Gender × Meeting Combined MVPA Threshold. Outcome: Low Back Pain; * *p <* 0.05, Significant.

**Table 12:** Multivariable Logistic Regression Analysis Examining the Interaction Between Meeting the Moderate-Intensity Physical Activity Threshold and Sex on Low Back Pain.

		B	S.E.	Wald	df	aOR	95% CI		*p* Value
							Lower	Upper	
Gender	Female	Ref	Ref	Ref	Ref	Ref	Ref	Ref	Ref
	Male	−0.829	0.274	9.144	1	0.436	0.255	0.747	0.002 *
Age	19–29	Ref	Ref	Ref	Ref	Ref	Ref	Ref	Ref
	30–39	1.245	0.292	18.222	1	3.473	1.961	6.152	<0.001 *
	40–49	1.514	0.301	25.258	1	4.543	2.518	8.197	<0.001 *
	≥50	1.589	0.348	20.867	1	4.898	2.477	9.684	<0.001 *
BMI Category	Normal (18.5–24.9)	Ref	Ref	Ref	Ref	Ref	Ref	Ref	Ref
	Underweight (<18.5)	−1.055	0.665	2.520	1	0.348	0.095	1.281	0.112
	Overweight (25.0–29.9)	0.031	0.250	0.015	1	1.031	0.631	1.685	0.902
	Obese (≥30.0)	0.351	0.302	1.351	1	1.421	0.786	2.567	0.245
Meets Moderate PA Threshold	No	Ref	Ref	Ref	Ref	Ref	Ref	Ref	Ref
	Yes	−0.087	0.276	0.100	1	0.917	0.534	1.574	0.752
Interaction: Gender × Meeting Moderate PA Threshold	Female × No	Ref	Ref	Ref	Ref	Ref	Ref	Ref	Ref
	Male × Yes	−0.315	0.448	0.494	1	0.730	0.303	1.757	0.482

Predictors: Gender, Age, Body Mass Index (BMI), Meeting Moderate PA Threshold (≥150 min/week), Interaction: Gender × Meeting Moderate PA Threshold. Outcome: Low Back Pain; * *p <* 0.05, Significant.

**Table 13:** Multivariable Logistic Regression Analysis Examining the Interaction Between Meeting the Vigorous-Intensity Physical Activity Threshold and Sex on Low Back Pain.

		B	S.E.	Wald	df	aOR	95% CI		*p* Value
							Lower	Upper	
Gender	Female	Ref	Ref	Ref	Ref	Ref	Ref	Ref	Ref
	Male	−0.991	0.291	11.598	1	0.371	0.210	0.657	<0.001 *
Age	19–29	Ref	Ref	Ref	Ref	Ref	Ref	Ref	Ref
	30–39	1.239	0.290	18.235	1	3.453	1.955	6.100	<0.001 *
	40–49	1.542	0.303	25.988	1	4.674	2.584	8.457	<0.001 *
	≥50	1.591	0.352	20.440	1	4.909	2.463	9.786	<0.001 *
BMI Category	Normal (18.5–24.9)	Ref	Ref	Ref	Ref	Ref	Ref	Ref	Ref
	Underweight (<18.5)	−1.044	0.664	2.473	1	0.352	0.096	1.293	0.116
	Overweight (25.0–29.9)	0.046	0.251	0.034	1	1.047	0.641	1.712	0.854
	Obese (≥30.0)	0.348	0.302	1.327	1	1.416	0.784	2.557	0.249
Meets Vigorous PA Threshold	No	Ref	Ref	Ref	Ref	Ref	Ref	Ref	Ref
	Yes	−0.125	0.272	0.210	1	0.883	0.519	1.503	0.647
Interaction: Gender × Meeting Vigorous PA Threshold	Female × No	Ref	Ref	Ref	Ref	Ref	Ref	Ref	Ref
	Male × Yes	0.144	0.426	0.115	1	1.155	0.501	2.663	0.735

Predictors: Gender, Age, Body Mass Index (BMI), Meeting Vigorous PA Threshold (≥75 min/week), Interaction: Gender × Meeting Vigorous PA Threshold. Outcome: Low Back Pain; * *p <* 0.05, Significant.

**Table 14:** Multivariable Logistic Regression Analysis Assessing the Interaction Between Sex and Meeting the Muscle-Strengthening Activity Frequency Threshold in Relation to Low Back Pain.

		B	S.E.	Wald	df	aOR	95% CI		*p* Value
							Lower	Upper	
Gender	Female	Ref	Ref	Ref	Ref	Ref	Ref	Ref	Ref
	Male	−0.999	0.272	13.445	1	0.368	0.216	0.628	<0.001 *
Age	19–29	Ref	Ref	Ref	Ref	Ref	Ref	Ref	Ref
	30–39	1.303	0.293	19.723	1	3.682	2.071	6.545	<0.001 *
	40–49	1.527	0.303	25.307	1	4.603	2.539	8.343	<0.001 *
	≥50	1.605	0.349	21.099	1	4.978	2.510	9.874	<0.001 *
BMI Category	Normal (18.5–24.9)	Ref	Ref	Ref	Ref	Ref	Ref	Ref	Ref
	Underweight (<18.5)	−1.196	0.669	3.201	1	0.302	0.082	1.121	0.074
	Overweight (25.0–29.9)	0.032	0.251	0.016	1	1.033	0.631	1.690	0.898
	Obese (≥30.0)	0.329	0.304	1.169	1	1.389	0.766	2.519	0.280
Meets MSA Frequency Threshold	No	Ref	Ref	Ref	Ref	Ref	Ref	Ref	Ref
	Yes	−0.633	0.306	4.287	1	0.531	0.292	0.967	0.038 *
Interaction: Gender × Meeting MSA Frequency Threshold	Female × No	Ref	Ref	Ref	Ref	Ref	Ref	Ref	Ref
	Male × Yes	0.310	0.467	0.441	1	1.364	0.546	3.407	0.507

Predictors: Gender, Age, Body Mass Index (BMI), Meeting Muscle-Strengthening Activity (MSA) Frequency Threshold (≥2 Days/Week), Interaction: Gender × Meeting MSA Frequency Threshold. Outcome: Low Back Pain; * *p <* 0.05, Significant.

**Table 15:** Multivariable Logistic Regression Analysis of Factors Associated with Low Back Pain According to Continuous Combined Physical Activity.

		B	S.E.	Wald	df	aOR	95% CI		*p* value
							Lower	Upper	
Gender	Female	Ref	Ref	Ref	Ref	Ref	Ref	Ref	Ref
	Male	−0.932	0.227	16.794	1	0.394	0.252	0.615	<0.001 *
Age	19–29	Ref	Ref	Ref	Ref	Ref	Ref	Ref	Ref
	30–39	1.234	0.291	18.029	1	3.437	1.944	6.076	<0.001 *
	40–49	1.536	0.303	25.755	1	4.648	2.568	8.414	<0.001 *
	≥50	1.591	0.348	20.884	1	4.908	2.481	9.711	<0.001 *
BMI Category	Normal (18.5–24.9)	Ref	Ref	Ref	Ref	Ref	Ref	Ref	Ref
	Underweight (<18.5)	−1.059	0.665	2.534	1	0.347	0.094	1.278	0.111
	Overweight (25.0–29.9)	0.044	0.250	0.030	1	1.045	0.639	1.706	0.862
	Obese (≥30.0)	0.357	0.301	1.399	1	1.428	0.791	2.579	0.237
Continuous Combined PA		−0.016	0.039	0.164	1	0.985	0.913	1.062	0.686

Predictors: Gender, Age, Body Mass Index (BMI), Log-transformed Total Combined Physical Activity (PA); Outcome: Low Back Pain; * *p <* 0.05, Significant.

**Table 16:** Multivariable Logistic Regression Analysis of Factors Associated with Low Back Pain According to Continuous Moderate Physical Activity.

		B	S.E.	Wald	df	aOR	95% CI		*p* Value
							Lower	Upper	
Gender	Female	Ref	Ref	Ref	Ref	Ref	Ref	Ref	Ref
	Male	−0.944	0.228	17.124	1	0.389	0.249	0.609	<0.001 *
Age	19–29	Ref	Ref	Ref	Ref	Ref	Ref	Ref	Ref
	30–39	1.219	0.291	17.599	1	3.385	1.915	5.983	<0.001 *
	40–49	1.516	0.301	25.401	1	4.554	2.525	8.211	<0.001 *
	≥50	1.586	0.347	20.901	1	4.885	2.475	9.643	<0.001 *
BMI Category	Normal (18.5–24.9)	Ref	Ref	Ref	Ref	Ref	Ref	Ref	Ref
	Underweight (<18.5)	−1.082	0.665	2.643	1	0.339	0.092	1.249	0.104
	Overweight (25.0–29.9)	0.053	0.250	0.044	1	1.054	0.645	1.722	0.834
	Obese (≥30.0)	0.363	0.302	1.450	1	1.438	0.796	2.597	0.229
Continuous Moderate PA		−0.050	0.042	1.395	1	0.951	0.876	1.033	0.238

Predictors: Gender, Age, Body Mass Index (BMI), Log-transformed Moderate Physical Activity (PA); Outcome: Low Back Pain; * *p <* 0.05, Significant.

**Table 17:** Multivariable Logistic Regression Analysis of Factors Associated with Low Back Pain According to Continuous Vigorous Physical Activity.

		B	S.E.	Wald	df	aOR	95% CI		*p* Value
							Lower	Upper	
Gender	Female	Ref	Ref	Ref	Ref	Ref	Ref	Ref	Ref
	Male	−0.930	0.227	16.745	1	0.395	0.253	0.616	<0.001 *
Age	19–29	Ref	Ref	Ref	Ref	Ref	Ref	Ref	Ref
	30–39	1.250	0.292	18.350	1	3.490	1.970	6.183	<0.001 *
	40–49	1.559	0.303	26.516	1	4.755	2.627	8.607	<0.001 *
	≥50	1.612	0.351	21.049	1	5.011	2.517	9.975	<0.001 *
BMI Category	Normal (18.5–24.9)	Ref	Ref	Ref	Ref	Ref	Ref	Ref	Ref
	Underweight (<18.5)	−1.039	0.664	2.447	1	0.354	0.096	1.300	0.118
	Overweight (25.0–29.9)	0.036	0.250	0.021	1	1.037	0.635	1.694	0.885
	Obese (≥30.0)	0.350	0.301	1.346	1	1.419	0.786	2.560	0.246
Continuous Vigorous PA		0.004	0.041	0.010	1	1.004	0.926	1.089	0.919

Predictors: Gender, Age, Body Mass Index (BMI), Log-transformed Vigorous Physical Activity (PA); Outcome: Low Back Pain; * *p <* 0.05, Significant.

**Table 18:** Low Back Pain by Physical Activity and Muscle-Strengthening Activity Category, With Crude and Adjusted Odds Ratios (*N* = 449).

Exposure	Category	*n*	LBP, *n* (%)	Crude OR (95% CI)	aOR (95% CI) ^a^
Combined MVPA threshold	Not met	194	109 (56.2%)	Ref	Ref
	Met	255	130 (51.0%)	0.811 (0.557–1.180)	0.904 (0.599–1.364)
Moderate-intensity PA threshold	Not met	291	163 (56.0%)	Ref	Ref
	Met	158	76 (48.1%)	0.728 (0.493–1.073)	0.813 (0.532–1.244)
Vigorous-intensity PA threshold	Not met	258	145 (56.2%)	Ref	Ref
	Met	191	94 (49.2%)	0.755 (0.519–1.099)	0.935 (0.618–1.416)
MSA frequency threshold	Not met	322	183 (56.8%)	Ref	Ref
	Met	127	56 (44.1%)	0.599 (0.396–0.906)	0.606 (0.385–0.954)
MSA frequency	0 days/week	259	150 (57.9%)	Ref	Ref
	1 day/week	63	33 (52.4%)	0.799 (0.460–1.389)	0.949 (0.515–1.748)
	≥2 days/week	127	56 (44.1%)	0.573 (0.373–0.880)	0.600 (0.376–0.959)
MSA frequency, contrast ^b^	≥2 vs. 1 day/week			0.717 (0.391–1.314)	0.632 (0.323–1.239)

OR, odds ratio; aOR, adjusted odds ratio; CI, confidence interval; LBP, low back pain; MVPA, moderate-to-vigorous physical activity; MSA, muscle-strengthening activity; PA, physical activity; Ref, reference category. Thresholds: combined MVPA, ≥150 moderate-equivalent min/week; moderate-intensity PA, ≥150 min/week; vigorous-intensity PA, ≥75 min/week; MSA frequency, ≥2 days/week. Walking is not counted in any PA threshold. ^a^ Adjusted for age, sex, and BMI category (Table 6, Table 7, Table 8, Table 9 and Table 10). ^b^ Same model as the row above, refitted with 1 day/week as the reference category.

## Data Availability

The data presented in this study are available on reasonable request from the corresponding author. The data are not publicly available because of privacy and ethical restrictions.

## Supplementary Materials

The following supporting information can be downloaded at https://www.mdpi.com/article/10.3390/healthcare14213571/s1. Table S1: Sensitivity analyses: adjusted odds ratios (95% CI) for chronic low back pain.

## Abbreviations

The following abbreviations are used in this manuscript:

aOR	Adjusted odds ratio
BMI	Body mass index
CI	Confidence interval
IPAQ-SF	International Physical Activity Questionnaire-Short Form
LBP	Low back pain
MSA	Muscle-strengthening activity
MVPA	Moderate-to-vigorous physical activity
NHANES	National Health and Nutrition Examination Survey
NIH	National Institutes of Health
OR	Odds ratio
PA	Physical activity
SAR	Saudi riyal
SD	Standard deviation
STROBE	Strengthening the Reporting of Observational Studies in Epidemiology
VIF	Variance inflation factor
WHO	World Health Organization
