# Supplementary material for: Low Back Pain, Physical Activity, and Muscle-Strengthening Activity Among Saudi Adults: A Cross-Sectional Study
Source: Healthcare (Basel). 2026 Oct 22;14(21):3571. doi: 10.3390/healthcare14213571 (PMC13763508; doi:10.3390/healthcare14213571)
Supplement: Supplementary file 1 [file healthcare-14-03571-s001.zip › healthcare-4611586-supplementary.pdf]

Supplementary Materials

Low Back Pain, Physical Activity, and Muscle-Strengthening Activity Among Saudi Adults: A Cross-Sectional Study

Table S1. Sensitivity analyses: adjusted odds ratios (95% CI) for chronic low back pain.

Exposure	Main model[a]	Plus sociodemographic covariates[b]	LBP <3 months kept as non-cases[c]
Combined MVPA threshold	0.904 (0.599–1.364)	0.951 (0.621–1.455)	0.801 (0.549–1.167)
Moderate-intensity PA threshold	0.813 (0.532–1.244)	0.838 (0.543–1.294)	0.753 (0.511–1.108)
Vigorous-intensity PA threshold	0.935 (0.618–1.416)	0.953 (0.622–1.460)	1.031 (0.705–1.507)
MSA frequency threshold	0.606 (0.385–0.954)	0.567 (0.355–0.905)	0.630 (0.415–0.957)
MSA 1 vs 0 days/week	0.949 (0.515–1.748)	0.954 (0.502–1.815)	1.000 (0.570–1.756)
MSA ≥2 vs 0 days/week	0.600 (0.376–0.959)	0.562 (0.347–0.911)	0.630 (0.410–0.969)
MSA ≥2 vs 1 day/week	0.632 (0.323–1.239)	0.589 (0.292–1.187)	0.630 (0.339–1.171)

[a]Adjusted for age, sex, and BMI category (N=449).

[b]Main model plus education, employment status, marital status, region, and household income (N=449).

[c]Main model with the 81 respondents with LBP lasting <3 months returned to the sample and classified as not having chronic LBP (N=530).

CI, confidence interval; LBP, low back pain; MVPA, moderate-to-vigorous physical activity; MSA, muscle-strengthening activity; PA, physical activity. Each exposure meeting its threshold is compared with not meeting it. Walking is not counted in any PA threshold.
